# Long-Term Implementation and Effectiveness of a Quality Improvement Intervention for Myocardial Infarction in Tanzania

**DOI:** 10.5334/aogh.5134

**Published:** 2026-07-02

**Authors:** Claire Wang, Francis M Sakita, Tarun Prakash, Victoria Dronzek, Thierry Kabwe, Zebadia Martin, Theresia Joachim, Zoë Wohlgenant, Arthi Vaidyanathan, Frida M Shayo, Ally M Akrabi, Gloria J Manyangu, Nathan M Thielman, Janet P Bettger, Julian Hertz

**Affiliations:** 1 Duke University School of Medicine, Durham, North Carolina, USA; 2 Kilimanjaro Christian Medical Centre, Moshi, Tanzania; 3 KCMC University, Moshi, Tanzania; 4 Tulane University School of Medicine, New Orleans, Louisiana, USA; 5 Department of Emergency Medicine, Duke University, Durham, North Carolina, USA; 6 Department of Emergency Medicine, Kilimanjaro Christian Medical Centre, Moshi, Tanzania; 7 Department of Emergency Medicine, Bugando Medical Centre, Mwanza, Tanzania; 8 Bugando Medical Centre, Mwanza, Tanzania; 9 Duke Global Health Institute, Duke University, Durham, North Carolina, USA; 10 Division of Infectious Diseases, Department of Medicine, Duke University Medical Center, Durham, North Carolina, USA; 11 Department of Physical Medicine and Rehabilitation, University of North Carolina School of Medicine, North Carolina, USA

**Keywords:** myocardial infarction, implementation science, quality improvement, Tanzania, sub-Saharan Africa, emergency care

## Abstract

*Background:* In Tanzania, acute myocardial infarction (AMI) is underdiagnosed, and uptake of evidence-based care remains limited. The Multicomponent Intervention to Improve Myocardial Infarction Care in Tanzania (MIMIC) increased evidence-based AMI care in a pilot trial conducted in a Tanzanian emergency department (ED). Whether MIMIC was sustained as implemented and its gains persisted after the pilot is unknown.

*Objectives:* To evaluate the long-term fidelity, penetration, and effectiveness of MIMIC one year after the pilot trial.

*Methods:* We conducted a sequential cohort study in a Tanzanian ED, enrolling adults with chest pain or dyspnea during the pilot period (September 2023–August 2024) and post-pilot period (September 2024–August 2025). Pre-intervention data (February–August 2023) served as supplemental baseline. Outcomes included fidelity and penetration of MIMIC components and 11 AMI care metrics. Proportions were compared using Pearson’s χ² tests and odds ratios (ORs) with 95% confidence intervals (CIs).

*Findings:* Of 260 post-pilot participants, 29 had AMI. Fidelity and penetration of MIMIC components were similar to pilot levels. Electrocardiogram (ECG) uptake remained high (~90% in both periods). Cardiac biomarker testing was lower post-pilot (64.0% [*n* = 203, excluding stock-out periods] vs 78.0%, OR 0.50, 95% CI 0.35–0.72; *P* < 0.001), coinciding with reagent stock-outs, but exceeding pre-intervention baseline (41.4%). Among AMI participants, treatment with aspirin, clopidogrel, heparin, and statins in the ED was comparable between pilot and post-pilot periods. Compared with the pre-intervention baseline, uptake of diagnostic testing and evidence-based therapies was substantially higher, including a >5-fold increase in 30-day antiplatelet use (10% vs 52%; OR 9.54, 95% CI 2.49–46.46; *P* < 0.001).

*Conclusions:* One year after implementation, MIMIC demonstrated sustained fidelity, penetration, and clinical effectiveness. AMI care remained substantially improved compared with pre-intervention baseline, suggesting that a pragmatic, workflow-integrated intervention can achieve durable gains in AMI care in a resource-limited ED.

## Introduction

Acute myocardial infarction (AMI) is a leading and rapidly growing cause of cardiovascular morbidity and mortality across sub-Saharan Africa (SSA) [1, 2]. As epidemiologic shifts lead to a growing burden of cardiovascular disease across the region [3–5], uptake of evidence-based AMI care remains limited and outcomes are suboptimal [6–9]. In northern Tanzania, 30-day mortality following AMI approaches 43% [10], and one-year mortality exceeds 50%—among the highest rates reported globally [11]. Early aspirin administration reduces short-term mortality by nearly 25% [12], and timely electrocardiogram (ECG) and biomarker testing are essential for rapid diagnosis and initiation of therapy [13]. Yet, these critical interventions are often delayed or omitted, resulting in missed opportunities for life-saving care [14].

At Kilimanjaro Christian Medical Centre (KCMC), a tertiary referral hospital in northern Tanzania, prior work identified important opportunities to strengthen emergency AMI care. In a prospective cohort of adults with chest pain or dyspnea, only 55% received an ECG, 41% underwent troponin testing, and only one-third received both [15]. Among patients ultimately diagnosed with AMI, just 34% were treated with aspirin [15]. These gaps, driven by low clinical suspicion, lack of standardized protocols, and resource constraints [16–19], drew attention to the need for feasible, contextually appropriate strategies to strengthen emergency cardiovascular care in low-resource settings [11].

To address these gaps, we developed the Multicomponent Intervention to Improve Myocardial Infarction Care (MIMIC), an emergency department (ED)-based quality improvement intervention and the first of its kind for AMI in Tanzania. MIMIC comprises five core components: provider training, clinical champions, color-coded triage tools, pocket reference cards, and patient education materials [18]. A one-year, single-arm pilot implementation trial evaluated the MIMIC intervention at KCMC [20]. The results of the trial have been previously published [20]; briefly, fidelity to the intervention components was high, with the exception of patient educational pamphlets [21]. Penetration of clinician-facing components was also strong, reflecting consistent integration into routine ED workflows [21]. These implementation outcomes were accompanied by significant improvements in AMI care delivery. Following implementation, uptake of ECG and cardiac biomarker testing increased among adults presenting with chest pain or dyspnea, and administration of evidence-based therapies such as aspirin, clopidogrel, and heparin increased among patients with AMI [20]. Concurrent evaluations demonstrated high provider-reported acceptability and feasibility and strong perceptions of normalization and perceived capacity for sustainability [22, 23]. Despite these gains, whether they would persist in routine practice beyond the trial remained unknown. In SSA, fewer than 40% of quality improvement interventions are sustained after trials or external funding conclude, resulting in poor long-term uptake [24]. This lack of continuity limits the impact of implementation research, eroding gains realized in initial studies and leading to inefficient use of both financial and human resources [24, 25]. To evaluate whether the clinical and implementation gains achieved during the MIMIC pilot trial were sustained in routine practice, we conducted a prospective, observational follow-up study at KCMC one year after the pilot’s conclusion.

This study evaluated the durability of MIMIC by assessing intervention fidelity, ED performance, and 30-day outcomes across 11 key AMI care metrics. The results are expected to inform strategies for sustaining implementation of evidence-based interventions for emergency cardiovascular care in similar resource-limited settings.

## Methods

### Setting

This study was conducted in the ED of KCMC, a 700-bed tertiary referral hospital in Moshi, northern Tanzania, serving an estimated 11 million people. The ED is staffed 24 hours per day by general physicians, emergency medicine-trained specialists, and registered nurses. During the study period, KCMC did not have a cardiologist or the capacity for percutaneous coronary intervention or cardiac surgery but maintains essential diagnostic and therapeutic resources for AMI, including ECG machines, cardiac biomarker testing, echocardiography, and medications such as aspirin, clopidogrel, beta-blockers, nitrates, heparin, statins, and thrombolytics.

### MIMIC intervention

The MIMIC intervention was designed to improve early AMI recognition and evidence-based management in a resource-limited ED. It consists of five integrated components: (1) red “AMI Suspect” triage cards placed by nurses on stretchers of patients presenting with chest pain or dyspnea to prompt physicians to consider the diagnosis of AMI, (2) pocket reference cards summarizing diagnostic and treatment steps for ED clinicians, (3) a mandatory web-based training module for all ED staff, (4) patient education materials, including pamphlets and visual displays, and (5) designated physician and nurse champions responsible for reinforcing adherence, conducting informal audits, and overseeing intervention implementation. The full MIMIC intervention has been previously published [18].

### Study design and timeline

This study evaluated MIMIC uptake and AMI clinical care during the 12 months following the pilot trial, from September 1, 2024, to August 31, 2025. The previously published pilot trial period was from September 1, 2023, to August 31, 2024. Prior to the initial pilot trial, pre-pilot baseline performance data were collected during a seven-month pre-pilot period. These periods are referred to throughout the manuscript as the pre-pilot baseline (February 1–August 31, 2023), pilot trial (September 1, 2023–August 31, 2024), and post-pilot period (September 1, 2024–August 31, 2025), respectively.

The primary analysis compared ED performance metrics during the post-pilot period with the initial pilot trial period to assess the sustainability of improvements in AMI care delivery. The secondary analysis compared the post-pilot period with the pre-pilot baseline to describe the persistence of clinical benefit from usual care. Implementation fidelity was assessed only during the pilot trial and post-pilot periods.

During both the pilot trial and post-pilot periods, implementation of the MIMIC was conducted entirely by the KCMC clinical team, without direct research team involvement for clinical implementation. Across all three study periods (pre-pilot baseline, pilot trial, and post-pilot period), research assistants (RAs) remained stationed in the ED to collect data but minimized interaction with clinical staff, relying primarily on the medical record to limit potential Hawthorne effects.

### Study population

Eligibility criteria were identical to the pilot trial. Adults aged ≥18 years presenting to the ED with chest pain or shortness of breath were eligible. Patients presenting with both symptoms simultaneously were eligible for inclusion. Presenting symptoms were determined by patient self-report, and this approach was consistent across all three study periods. Those with self-reported fever or trauma-related chest pain were excluded.

### Study procedures

Due to budgetary constraints, RA screening and enrollment hours differed between study periods. During the pilot trial, RAs screened patients from 8 a.m. to 11 p.m., seven days per week; during the post-pilot period, screening and enrollment occurred from 9 a.m. to 5 p.m., six days per week. Patients presenting outside RA coverage hours received usual ED care but were not screened or enrolled for study participation. RAs obtained written informed consent from all eligible participants. Consent and questionnaire administration were conducted in parallel with, and did not precede, clinical care. RAs were trained to ensure that research procedures did not delay clinical assessment or treatment initiation. After enrollment, they administered a brief baseline questionnaire capturing demographics, comorbidities, and presenting symptoms. RAs directly observed ED care and abstracted diagnostic and treatment information from the medical record, including whether and when ECG and cardiac biomarker testing were performed, the results of all laboratory studies, all AMI-related medications administered, and the final documented ED or hospital diagnosis. ECG tracings were digitally captured and independently reviewed by two blinded adjudicators selected from a pool of four emergency physicians and one medical student to determine the presence of ST-elevation myocardial infarction (STEMI), such that no diagnosis depended on the interpretation of a single reader. The medical student had completed paramedic training that included formal instruction and supervised experience in ECG interpretation and STEMI recognition. This process was identical to that used in the pilot trial, with an updated pool of readers. Discrepancies were adjudicated by a third blinded physician. Thirty-day follow-up was conducted by telephone by the RAs, with home visits for participants unreachable by phone to ascertain vital status. AMI was defined as either STEMI, as determined by the ECG adjudication process described above, or non-ST-elevation myocardial infarction (NSTEMI), defined by a positive cardiac biomarker result with an abnormal delta troponin or a physician-documented diagnosis of NSTEMI in the medical record. A uniform AMI study definition was used across all study phases [20].

### Study measures

*Implementation fidelity* was assessed by the proportion of eligible opportunities in which each intervention component was delivered, mirroring the pilot trial approach [20]. RAs documented triage card placement and patient pamphlet distribution through direct observation, verified online module initiation using KCMC training logs, and recorded pocket card use through direct observation. Champion activity was tracked by whether AMI care audits were performed at least monthly.

*Penetration* was defined as the degree to which each component was integrated into routine care, operationalized as the proportion of discrete clinician-shifts in which the component was observed in use (for triage cards and pocket cards), the proportion of ED staff who completed the online module, and the proportion of AMI participants who reported reading the educational pamphlet at 30 days.

*ED performance and clinical outcomes* were evaluated using 11 key AMI care metrics: (1) ECG completion in the ED, (2) cardiac biomarker testing (troponin or CK-MB) in the ED, (3) identification of AMI per the study definition, (4) administration of aspirin to patients with AMI, (5) administration of clopidogrel or other P2Y12 inhibitor, (6) administration of heparin, (7) administration of a statin, (8) administration of thrombolytic therapy, (9) referral to a specialty cardiac center, (10) 30-day antiplatelet use, and (11) 30-day survival. All outcomes were defined and measured identically to the pilot trial to allow direct comparison, with three exceptions. First, because of periodic troponin assay stock-outs and the use of creatine kinase-MB (CK-MB) as an alternative marker during these intervals, the cardiac biomarker outcome was defined as completion of any cardiac biomarker test (troponin or CK-MB) in the ED. Second, the combined outcome of completion of both ECG and troponin testing, used in the pilot trial, was not evaluated in the post-pilot period. Third, 30-day antiplatelet use was included as a key AMI care metric in the present study; although this outcome was collected during the pilot trial [21], detailed comparative analyses have been reported separately.

### Statistical analysis

Patient characteristics, clinical performance metrics, and fidelity measures were summarized using descriptive statistics. Continuous variables are reported as means with standard deviations (SD) and compared using Welch’s *t*-tests. Categorical variables are presented as counts and percentages and compared using Pearson’s chi-squared or Fisher’s exact tests when expected cell counts were <5. For each clinical performance metric, the proportion of eligible patients achieving the outcome during the post-pilot period was compared with the initial pilot trial period. Odds ratios (ORs) with 95% confidence intervals (CIs) were calculated from contingency tables to quantify the relative likelihood of achieving each outcome. For each implementation outcome, we calculated the proportion of eligible patients or providers achieving the outcome, using component-specific denominators. Due to periodic stock-outs of cardiac biomarker assay reagents, patients presenting during documented stock-out intervals were excluded from the denominator for analyses of cardiac biomarker testing uptake. Analyses were complete case only; no imputation was performed for missing data. No a priori effect size assumptions or target sample size were specified, as the post-pilot period was intended to be descriptive rather than powered for inferential comparisons. All tests were two-tailed with a significance threshold of *P* < 0.05. Analyses were conducted using R version 4.5.1 (R Core Team, 2024). As a supplemental analysis, clinical performance for the 11 key AMI care metrics was compared between the initial pre-intervention baseline period and post-pilot period, using the same statistical methods described above.

### Ethics

Ethical approval was obtained from the Tanzania National Institute for Medical Research (NIMR/HQ/R.8a/Vol. IX/2436), Kilimanjaro Christian Medical Centre (Proposal 893), and the Duke Health Institutional Review Board (Pro00090902). All procedures adhered to the ethical principles outlined in the Declaration of Helsinki (2000 revision). Written informed consent was obtained from all participants prior to data collection. All materials were provided in English and Swahili to ensure comprehension, and participation was entirely voluntary, and participants were explicitly informed that their care would not be affected by their decision to participate. Participants could decline involvement or withdraw at any time without penalty.

## Results

**Figure 1:**
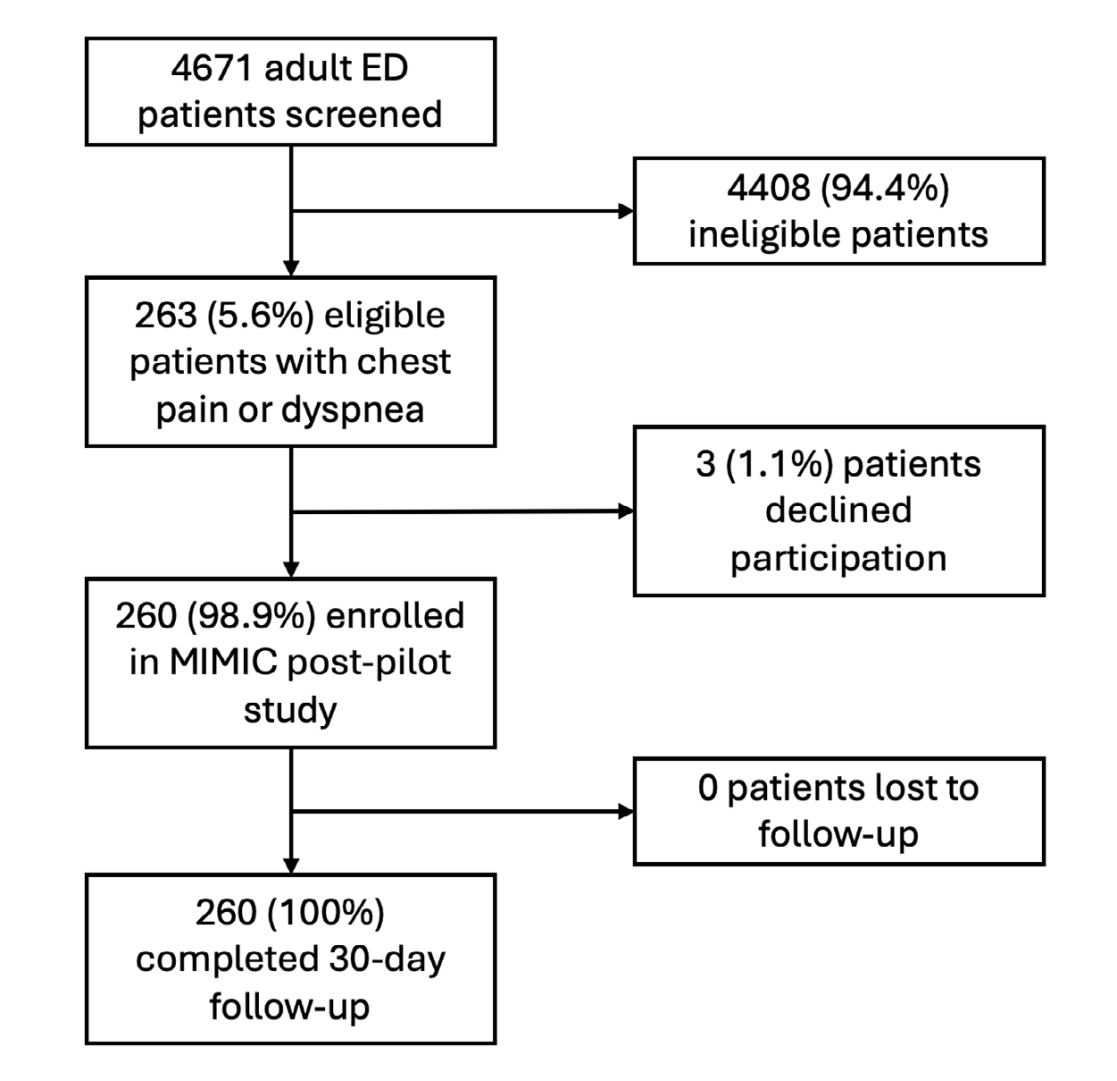
Participant flow diagram.

During the post-pilot period, a total of 4671 adult patients presenting to the KCMC ED were screened, of whom 263 (5.6%) had chest pain or dyspnea and were eligible for enrollment. A total of three (1.1%) eligible patients declined participation, and the remaining 260 (98.9%) provided consent and were enrolled (Figure 1). No enrolled participants were lost to follow-up.

Table 1 summarizes participant characteristics in the pilot and post-pilot cohorts. Slightly more than half of participants in each period were female (56.5% of post-pilot participants vs 52.7% of pilot participants; OR 1.17, 95% CI 0.86–1.59; *P* = 0.330). The mean (SD) age of participants was 62.8 (17.7) years in the post-pilot period and 62.4 (17.8) years in the pilot period (*P* = 0.759). Mean systolic blood pressure (BP) was similar between groups, while mean diastolic BP was slightly lower in the post-pilot group (78.9 vs 82.5 mmHg; *P* = 0.008). AMI was identified in 141 (24.4%) of 577 pilot participants and 29 (11.2%) of 260 post-pilot participants.

**Table 1:** Baseline characteristics of adult patients presenting to the KCMC ED with chest pain or shortness of breath in the pilot trial and post-pilot periods.

	PILOT TRIAL PARTICIPANTS (*N* = 577)		POST-PILOT PARTICIPANTS (*N* = 260)		OR (95% CI)	*P*
CHARACTERISTIC	*N*	(%)	*N*	(%)		
Sex						
Female	304	(52.7)	147	(56.5)	1.17 (0.86–1.59)	0.330
Male	273	(47.3)	113	(43.5)		
History of tobacco use	202	(35.0)	78	(30.0)	0.80 (0.58–1.09)	0.178
History of alcohol use	414	(71.8)	178	(68.5)	0.85 (0.61–1.19)	0.367
History of hypertension	373	(64.6)	165	(62.5)	0.91 (0.67–1.25)	0.588
History of diabetes	158	(27.4)	79	(29.9)	1.13 (0.81–1.58)	0.458
History of heart failure	137	(23.7)	79	(29.9)	1.37 (0.97–1.92)	0.062
History of prior MI	36	(6.2)	10	(3.8)	0.59 (0.26–1.24)	0.191
History of stroke	26	(4.5)	7	(2.7)	0.58 (0.21–1.39)	0.252
History of HIV	19	(3.3)	9	(3.5)	1.05 (0.41–2.48)	0.999
	**PILOT TRIAL PARTICIPANTS (*N* = 577)**		**POST-PILOT PARTICIPANTS (*N* = 260)**			***P***
	**MEAN**	**(SD)**	**MEAN**	**(SD)**		
Age (years)	62.4	(17.8)	62.8	(17.7)		0.759
Symptom duration prior to ED presentation (days)	4.9	(11.8)	6.5	(10.7)		0.077
Systolic BP (mmHg)	141.8	(32.6)	137.4	(30.9)		0.062
Diastolic BP (mmHg)	82.5	(20.1)	78.9	(17.1)		0.008*

*P < 0.05 using Welch’s *t*-test.

There were otherwise no significant differences in baseline characteristics between pilot and post-pilot participants, including history of tobacco use, hypertension, diabetes, prior myocardial infarction, stroke, heart failure, or HIV infection (Table 1).

### Fidelity

Fidelity to core components of the MIMIC intervention remained high during the post-pilot period (Table 2), with no significant differences in fidelity between the pilot and post-pilot periods. Champions were active throughout both study periods, conducting regular auditing and reinforcing adherence on at least a monthly basis. Fidelity to the pocket card component was excellent across both periods, with 100% of physicians observed to have brought their pocket cards to work on at least one occasion (21 of 21 post-pilot; 22 of 22 pilot). With regard to the online training module, a similar proportion of ED staff started the module in both periods, with 31 of 36 staff (86%) starting the module in the post-pilot period compared with 45 of 54 staff (83%) in the pilot period (OR 1.24, 95% CI 0.33–5.17; *P* = 0.776). For the triage card component, 189 (72.7%) of 260 post-pilot participants received an “AMI Suspect” triage card, compared with 453 (78.5%) of 577 eligible pilot participants (OR 0.73, 95% CI 0.51–1.04; *P* = 0.077). Fidelity to the patient educational pamphlet remained low in both periods, with 12 (41%) of 29 post-pilot AMI participants receiving a pamphlet compared with 53 (37.6%) of 141 AMI participants in the pilot period (OR 1.17, 95% CI 0.47–2.84; *P* = 0.834).

**Table 2:** Fidelity of implementation of the MIMIC intervention in the KCMC ED during the post-pilot period.

FIDELITY MEASURE	DENOMINATOR	PILOT TRIAL PARTICIPANTS (*N* = 577)			POST-PILOT PARTICIPANTS (*N* = 260)			OR (95% CI)	*P*
		TOTAL *N*	OBSERVED *N*	%	TOTAL *N*	OBSERVED *N*	%		
Proportion of participants with chest pain/dyspnea correctly flagged the “AMI Suspect” triage card	Patient participants with AMI symptoms	577	453	78.5%	260	189	72.7%	0.73 (0.51–1.04)	0.077
Proportion of physicians observed to have ever brought their pocket cards to work	ED physicians	22	22	100%	21	21	100%	—	—
Proportion of ED staff starting the online training module	Total number of ED physicians and nurses	54	45	83%	36	31	86%	1.24 (0.33–5.17)	0.776
Proportion of participants with AMI receiving the educational pamphlet in the ED	Total number of patient participants with confirmed AMI	141	53	37.6%	29	12	41%	1.17 (0.47–2.84)	0.834

### Penetration

Penetration of intervention components among target audiences is presented in Table 3. Pocket cards demonstrated the highest penetration, with pocket cards directly observed during 97.7% (1504/1539) of physician-shifts in the post-pilot period and 96.1% (1835/1910) in the pilot period (OR 1.76, 95% CI 1.17–2.64; *P* = 0.006), representing a small but statistically significant increase in the post-pilot period. Penetration of the online training module among ED staff was comparable across the two periods (86% in the post-pilot period vs 76% in the pilot period; OR 1.97, 95% CI 0.63–6.10; *P* = 0.237). Notably, in the pilot period, 4 of the 45 staff who initiated the training module did not complete it, whereas in the post-pilot period, all staff who initiated the module also completed it. Penetration of the “AMI Suspect” triage card among nurses working in triage was moderate across both periods but declined in the post-pilot period, observed during 60.0% (342/580) of triage-shifts in the post-pilot period and 67.2% (572/851) in the pilot period (OR 0.70, 95% CI 0.56–0.87; *P* = 0.002). Penetration of the educational pamphlets remained limited: among AMI participants who received a pamphlet and survived to 30 days, 6 (67%) of 9 post-pilot participants and 22 (56%) of 39 pilot participants reported reading the pamphlet (OR 1.53, 95% CI 0.28–10.84; *P* = 0.716).

**Table 3:** Penetration of implementation of the MIMIC intervention in the KCMC ED during the post-pilot period.

PENETRATION MEASURE	DENOMINATOR	PILOT TRIAL PERIOD			POST-PILOT PERIOD			OR (95% CI)	*P*
		TOTAL *N*	OBSERVED *N*	%	TOTAL *N*	OBSERVED *N*	%		
Proportion of nurses working in triage directly observed to use the “AMI Suspect” triage card	Discrete nurse triage-shift observations	851	572	67.2%	580	342	60.0%	0.70 (0.56–0.87)	0.002*
Proportion of physicians directly observed to have their pocket card at work	Discrete physician-shift observations	1910	1835	96.1%	1539	1504	97.7%	1.76 (1.17–2.64)	0.006*
Proportion of ED providers completing the online training module	All ED staff	54	41	76%	36	31	86%	1.97 (0.63–6.10)	0.237
Proportion of surviving participants with AMI who read the educational pamphlet within 30 days	AMI patients who received the educational pamphlet and survived to 30 days	39	22	56%	9	6	67%	1.53 (0.28–10.84)	0.716

*P < 0.05.

Table 4 compares uptake of diagnostic testing between the post-pilot and pilot periods. ECG use remained high across both periods: 232 (89.2%) of 260 post-pilot participants received an ECG, compared to 516 (89.4%) of 577 pilot participants (OR 0.98, 95% CI 0.60–1.64; *P* = 0.904). In contrast, cardiac biomarker testing was performed less frequently in the post-pilot period. Among eligible participants (*n* = 203, excluding those presenting during documented assay stock-outs), 130 (64.0%) underwent biomarker testing, compared to 450 (78.0%) of 577 pilot participants (OR 0.50, 95% CI 0.35–0.72; *P* < 0.001).

**Table 4:** Uptake of diagnostic testing and AMI case detection among adult patients presenting to the KCMC ED with chest pain or shortness of breath.

	PILOT TRIAL PARTICIPANTS (*N* = 577)		POST-PILOT PARTICIPANTS (*N* = 260)		OR (95% CI)	*P*
	*N*	(%)	*N*	(%)		
ECG obtained	516	(89.4)	232	(89.2%)	0.98 (0.60–1.64)	0.904
Cardiac biomarker obtained	450	(78.0)	130^a^	(64.0%)	0.50 (0.35–0.72)	<0.001*

*P < 0.05.

^a^Denominator (*N* = 203) excludes patients presenting during documented cardiac biomarker assay stock-outs.

Table 5 presents uptake of evidence-based AMI therapies among post-pilot and pilot participants with confirmed AMI. Aspirin use in the ED was similar across the two periods: 19 (66%) of 29 post-pilot participants received aspirin, compared to 101 (71.6%) of 141 pilot participants (OR 0.75, 95% CI 0.30–1.98; *P* = 0.509). Uptake of clopidogrel (59% vs 65.2%, OR 0.76, 95% CI 0.31–1.89; *P* = 0.532), heparin (31% vs 43.2%, OR 0.59, 95% CI 0.22–1.48; *P* = 0.301), and statin therapy (59% vs 46.8%, OR 1.61, 95% CI 0.67–3.98; *P* = 0.309) was also comparable between periods. Use of thrombolytic therapy (10% vs 9.9%, OR 1.05, 95% CI 0.18–4.14; *P* = 0.999) and referral to a cardiac center (17% vs 13.5%, OR 1.34, 95% CI 0.35–4.20; *P* = 0.566) did not differ significantly between periods. With 29 post-pilot AMI participants, these comparisons are underpowered for inferential testing; the observed effect estimates and their associated CIs and *P*-values should be interpreted as exploratory.

**Table 5:** Uptake of evidence-based AMI therapy during and after the MIMIC intervention pilot trial in a Tanzanian ED.

	PILOT TRIAL AMI PARTICIPANTS (*N* = 141)^20^		POST-PILOT AMI PARTICIPANTS (*N* = 29)		OR (95% CI)	P*
THERAPY	*N*	(%)	*N*	(%)		
Aspirin	101	(71.6)	19	(66)	0.75 (0.30–1.98)	0.509
Clopidogrel	92	(65.2)	17	(59)	0.76 (0.31–1.89)	0.532
Heparin	61	(43.2)	9	(31)	0.59 (0.22–1.48)	0.301
Statin	66	(46.8)	17	(59)	1.61 (0.67–3.98)	0.309
Thrombolytic	14	(9.9)	3	(10)	1.05 (0.18–4.14)	0.999
Referral to cardiac center	19	(13.5)	5	(17)	1.34 (0.35–4.20)	0.566

*P < 0.05.

Thirty-day outcomes among participants with confirmed AMI are shown in Table 6. At 30 days, 21 (72%) of 29 post-pilot participants were alive, compared to 90 (63.8%) of 141 pilot participants (OR 1.48, 95% CI 0.58–4.17; *P* = 0.521). There was a trend toward higher antiplatelet use at 30 days in the post-pilot cohort, although this difference was not statistically significant. Among those with 30-day follow-up, 15 (52%) of 29 post-pilot participants and 51 (36.2%) of 141 pilot participants were taking an antiplatelet at 30 days (OR 1.88, 95% CI 0.78–4.59; *P* = 0.144). As noted above, the small post-pilot AMI sample limits the inferential value of *P*-values; CIs and effect estimates should guide interpretation.

**Table 6:** Thirty-day outcomes among participants with confirmed AMI.

THIRTY-DAY OUTCOME	DENOMINATOR	PILOT TRIAL PARTICIPANTS (*N* = 141)			POST-PILOT PARTICIPANTS (*N* = 29)			OR (95% CI)	*P*
		TOTAL *N*	OBSERVED *N*	%	TOTAL *N*	OBSERVED *N*	%		
Proportion of participants with AMI alive at 30 days	Participants with confirmed AMI	141	90	63.8%	29	21	72%	1.48 (0.58–4.17)	0.521
Proportion of participants with AMI taking an antiplatelet at 30 days	Participants with confirmed AMI and 30-day follow-up	141	51	36.2%	29	15	52%	1.88 (0.78–4.59)	0.144

In supplemental analyses comparing the pre-pilot and post-pilot periods (Supplemental Tables 1–3), uptake of diagnostic testing and most AMI therapies was higher in the post-pilot period, with others showing nonsignificant upward trends. ECG testing increased from 152 (55.3%) of 275 pre-pilot participants to 232 (89.2%) of 260 post-pilot participants (OR 6.68, 95% CI 4.17–11.00; *P* < 0.001), and cardiac biomarker testing from 114 (41.4%) of 275 to 130 (64.0%) of 203 participants (OR 2.51, 95% CI 1.70–3.72; *P* < 0.001). Among participants with AMI, uptake of aspirin (34% vs 66%; OR 3.59, 95% CI 1.21–11.30; *P* = 0.015), clopidogrel (27% vs 59%; OR 3.78, 95% CI 1.26–12.03; *P* = 0.013), heparin (5% vs 31%; OR 8.50, 95% CI 1.55–88.29; *P* = 0.006), and statin therapy (24% vs 59%; OR 4.29, 95% CI 1.41–13.95; *P* = 0.006) all increased significantly in the post-pilot period. Use of thrombolytic therapy (2% vs 10%; OR 4.52, 95% CI 0.34–247.85; *P* = 0.300) and referral to a cardiac center (5% vs 17%; OR 3.98, 95% CI 0.59–44.92; *P* = 0.118) were numerically higher in the post-pilot period but did not reach statistical significance. At 30-day follow-up, 21 (72%) of the post-pilot participants with AMI were alive, compared to 25 (61%) of the pre-pilot participants with AMI (OR 1.67, 95% CI 0.54–5.46; *P* = 0.444). The proportion of AMI participants taking an antiplatelet at 30 days increased from 4 (10%) of 41 pre-pilot participants to 15 (52%) of 29 post-pilot participants (OR 9.54, 95% CI 2.49–46.46; *P* < 0.001).

## Discussion

To our knowledge, this is the first study to evaluate the longer-term durability of an intervention to improve uptake of evidence-based AMI care in SSA. One year after completion of the MIMIC pilot trial, most early gains in diagnostic testing and initiation of evidence-based therapies were maintained under routine clinical conditions. Fidelity and penetration of intervention components remained stable, and performance across nearly all AMI care metrics remained significantly higher than pre-intervention baseline levels. Multisite cluster-randomized trials will be important to determine whether MIMIC’s effects on AMI care are generalizable and sustainable at scale.

Fidelity and penetration during the follow-up period were largely unchanged from the pilot period, suggesting long-term sustainment of the MIMIC intervention. Champion activity remained stable, and fidelity to the pocket card and online training components was high, with all physicians bringing their pocket cards to work at least once and most initiating the training module. Fidelity to components requiring additional steps during triage, including the AMI Suspect triage prompt and patient education pamphlets, remained modest, reflecting the influence of workflow demands during high-acuity encounters. Penetration patterns similarly mirrored those observed in the pilot trial: physician-facing components demonstrated the highest uptake, with pocket card use increasing slightly and approaching near-universal adoption, whereas nurse-led components showed more variable use and the AMI Suspect triage prompt declined slightly. Persistently high ECG uptake despite lower triage prompt use suggests that clinicians internalized core AMI recognition heuristics over time, maintaining diagnostic behavior even when visible prompts were inconsistently used. These patterns align closely with our CSAT and NoMAD findings, which captured ED clinicians’ perceptions of sustainability capacity and normalization [22]. Clinicians reported high perceived organizational capacity to sustain MIMIC and strong normalization into routine workflow, with high scores in domains related to clinical benefit, workflow compatibility, and engagement indicating that MIMIC was viewed as valuable, aligned with clinical priorities, and well-integrated into everyday care [22]. The convergence between observed behavior and provider-reported perceptions strengthens confidence that core MIMIC practices have become embedded within routine emergency care at KCMC. Notably, of the 35 clinicians working in the ED at the end of follow-up, 29 had participated in the initial implementation and six were newly hired, yet performance remained stable, further supporting that MIMIC-related practices persisted despite staff turnover. This level of sustainment contrasts with broader evidence that long-term maintenance of quality-improvement efforts is often limited; two recent reviews of implementation studies found that only about 60% of sites sustained even one program component after implementation and fewer than half demonstrated high-fidelity sustainment at 12 months or longer [26, 27]. In our study, core MIMIC components continued to be used routinely in the KCMC ED, supporting the intervention’s durability under real-world conditions.

In this context of sustained implementation of MIMIC, gains in diagnostic testing and uptake of evidence-based therapies were also maintained during the post-pilot period. ECG uptake remained high and unchanged compared with the pilot period, with approximately 90% of eligible patients receiving an ECG in both periods. Cardiac biomarker testing declined relative to the pilot period, coinciding with recurrent reagent stock-outs; however, biomarker use remained substantially higher than the pre-intervention baseline. These sustained improvements reflect MIMIC’s co-designed strategies, which directly targeted barriers to AMI recognition and treatment previously documented in northern Tanzania. Earlier qualitative work showed that AMI under-diagnosis commonly arose from low clinical suspicion, discomfort interpreting ECGs and troponins, and inconsistent prioritization of chest pain evaluation, even when diagnostic tools were available [9, 10, 17, 19]. MIMIC addressed these gaps by pairing focused training in AMI diagnosis with simple clinical prompts, including triage cards for nurses and pocket cards for physicians [18]. The continued use of these components during the post-pilot period suggests that locally developed, workflow-aligned tools can become embedded in routine practice and may support improved AMI case identification in similar settings across SSA. The decline in cardiac biomarker testing in the post-pilot period, however, is notable, and may be related to supply shortages. Providers may be less likely to order tests that are inconsistently available; this observation highlights the impact of resource limitations on sustainment of quality improvement interventions in settings like ours. Notably, AMI prevalence fell markedly between the pilot and post-pilot periods (24.4% vs 11.2%). This change coincides with the decline in biomarker testing and raises the possibility that a proportion of NSTEMI cases may not have been identified during the post-pilot period. Because NSTEMI diagnosis relies on biomarker evidence, patients who were not tested during shortage periods may have gone undetected, even if they received appropriate care. Accordingly, observed changes in AMI prevalence and case identification should be interpreted cautiously.

Uptake of evidence-based therapies was similar to pilot-period levels overall, with a trend toward increased statin use. Thrombolytic use and referral to percutaneous coronary intervention (PCI)-capable facilities also increased modestly but did not reach statistical significance, likely due to the small number of eligible patients. Sample sizes were likely too small to detect differences in 30-day mortality, although there was a nonsignificant trend toward increased survival during the post-pilot period. Thirty-day antiplatelet use trended higher, suggesting improvement in early post-discharge management. Notably, roughly one-third of patients with AMI still did not receive aspirin in the ED. Determining whether these omissions reflect contraindications, delayed recognition, or modifiable practice patterns could inform future refinement of MIMIC.

Comparisons to the pre-intervention period further underscore the durability of MIMIC’s impact. Relative to the pre-intervention baseline, both diagnostic testing and evidence-based treatment remained substantially higher during the post-pilot period, including a more than fivefold increase in 30-day antiplatelet use. The persistence of these gains is consistent with findings from major acute coronary syndrome quality-improvement trials. In the ACS-QUIK trial in India [28] and the BRIDGE-ACS trial in Brazil [29], interventions increased uptake of guideline-recommended therapies, although both trials were conducted in comparatively more resource-replete hospital systems. A systematic review of ACS quality-improvement programs in low- and middle-income countries reported modest improvements, typically in the range of 3% to 10%, in key in-hospital and discharge therapies [30]. While direct comparison is limited by differences in design and sample size, the consistency of improvement across studies supports that structured, workflow-aligned quality-improvement interventions can strengthen AMI care in resource-constrained settings.

This study has several strengths. The intervention was implemented entirely by local ED clinicians, strengthening real-world feasibility and external validity. AMI diagnoses were defined using standardized, guideline-concordant criteria [13], and 30-day follow-up was achieved for all patients. The study design incorporated both pilot-post and pre-post comparisons, providing complementary perspectives on performance over time. Objective performance data also aligned with earlier MIMIC studies demonstrating high acceptability and feasibility [23], which strengthens confidence in the intervention’s long-term durability. Several limitations should be considered. This was a single-site study, which may limit generalizability. Differences in sample sizes across comparison periods may have reduced statistical precision. In addition, some penetration measures were derived from repeated clinician-shift observations that may be correlated within clinicians; treating observations as independent may slightly underestimate variance, although effect estimates were directionally consistent and differences were small in magnitude. Enrollment hours were more limited during the post-pilot period, which may have affected representativeness if intervention delivery varied by time of day or day of week. Reduced RA coverage during the post-pilot period may have introduced selection bias. Prior studies have demonstrated circadian variation in AMI presentation, with a peak frequency of presentation during morning hours that partially fell outside post-pilot enrollment hours [31]. As a result, some AMI cases may have been missed, potentially contributing to an underestimation of AMI prevalence during the post-pilot period. Because enrollment was restricted to daytime hours, the post-pilot cohort may have overrepresented patients with delayed or subacute presentations and underrepresented patients presenting acutely overnight or in the early morning, potentially affecting cohort representativeness. As a pre-post analysis, findings may be influenced by unmeasured temporal trends, although no major secular changes in AMI care at KCMC were identified during the study period. Recurrent stock-outs of cardiac biomarker reagents limited completeness of biomarker assessment and may have contributed to the underdiagnosis of NSTEMI, which requires biomarker confirmation for diagnosis. The observed decline in AMI prevalence during the post-pilot period may therefore reflect, in part, reduced diagnostic capacity rather than a true change in disease burden. Direct observation may have introduced Hawthorne effects, and patient self-report may be subject to recall or social-desirability bias, although these are unlikely to explain the consistent performance patterns observed across periods.

In conclusion, MIMIC represents a pragmatic, sustainable, and potentially scalable approach to strengthening AMI care in EDs in SSA. Future work should evaluate the intervention across multiple hospitals in Tanzania, refine components with lower uptake (such as triage prompts and patient education), and incorporate cost-effectiveness analyses to inform policy adoption. Qualitative inquiry with clinicians and administrators will be important to clarify sustainability mechanisms and identify opportunities for workflow optimization. Given the high burden of AMI mortality in SSA and limited access to cardiology services, workflow-integrated interventions such as MIMIC may offer a feasible approach to improving early AMI recognition and evidence-based management in resource-limited settings.

## Additional Files

The additional files for this article can be found as follows:

Supplementary Table 1. Uptake of diagnostic testing and AMI case detection among adult patients presenting to the KCMC ED with chest pain or shortness of breath, comparing pre-pilot and post-pilot periods.

Supplementary Table 2. Uptake of evidence-based AMI therapy before and after the MIMIC intervention pilot trial in a Tanzanian emergency department.

Supplementary Table 3. Thirty-day outcomes among participants with confirmed AMI before and after the MIMIC intervention pilot trial in a Tanzanian emergency department.

## Study Data

All authors had access to the study data and had a role in writing the manuscript.
