# Supplementary material for: Long-Term Implementation and Effectiveness of a Quality Improvement Intervention for Myocardial Infarction in Tanzania
Source: Ann Glob Health. 2026 Jul 2;92(1):61. doi: 10.5334/aogh.5134 (PMC13330850; doi:10.5334/aogh.5134)
Supplement: Supplementary Table 1. — Uptake of diagnostic testing and AMI case detection among adult patients presenting to the KCMC ED with chest pain or shortness of breath, comparing pre-pilot and post-pilot periods. [file agh-92-1-5134-s1.pdf]

Supplemental Table 1.

Uptake of diagnostic testing and AMI case detection among adult patients presenting to the KCMC ED with chest pain or shortness of breath, comparing pre-pilot and post-pilot periods

	Pre-pilot participants (N=275)		Post-pilot participants (N=260)		Odds ratio (95% CI)	*p*
	n	(%)	n	(%)		
ECG obtained	152	(55.3%)	232	(89.2%)	6.68 (4.17-11.00)	<0.001*
Cardiac biomarker obtained	114	(41.4%)	130[a]	(64.0%)	2.51 (1.70-3.72)	<0.001*

[a] Denominator (N = 203) excludes patients presenting during documented cardiac biomarker assay stock-outs.

*$p < 0.05$
