# Supplementary material for: Long-Term Implementation and Effectiveness of a Quality Improvement Intervention for Myocardial Infarction in Tanzania
Source: Ann Glob Health. 2026 Jul 2;92(1):61. doi: 10.5334/aogh.5134 (PMC13330850; doi:10.5334/aogh.5134)
Supplement: Supplementary Table 2. — Uptake of evidence-based AMI therapy before and after the MIMIC intervention pilot trial in a Tanzanian emergency department. [file agh-92-1-5134-s2.pdf]

Supplemental Table 2.

Uptake of evidence-based AMI therapy before and after the MIMIC intervention pilot trial in a Tanzanian emergency department

	Pre-pilot participants (N=41)		Post-pilot participants (N=29)		Odds ratio (95% CI)	*p*
Therapy	n	(%)	n	(%)		
Aspirin	14	(34%)	19	(66%)	3.59 (1.21-11.30)	0.015*
Clopidogrel	11	(27%)	17	(59%)	3.78 (1.26-12.03)	0.013*
Heparin	2	(5%)	9	(31%)	8.50 (1.55-88.29)	0.006*
Statin	10	(24%)	17	(59%)	4.29 (1.41-13.95)	0.006*
Thrombolytic	1	(2%)	3	(10%)	4.52 (0.34-247.85)	0.300
Referral to cardiac center	2	(5%)	5	(17%)	3.98 (0.59-44.92)	0.118

*$p < 0.05$
