# Supplementary material for: Long-Term Implementation and Effectiveness of a Quality Improvement Intervention for Myocardial Infarction in Tanzania
Source: Ann Glob Health. 2026 Jul 2;92(1):61. doi: 10.5334/aogh.5134 (PMC13330850; doi:10.5334/aogh.5134)
Supplement: Supplementary Table 3. — Thirty-day outcomes among participants with confirmed AMI before and after the MIMIC intervention pilot trial in a Tanzanian emergency department. [file agh-92-1-5134-s3.pdf]

Supplemental Table 3.

Thirty-day outcomes among participants with confirmed AMI before and after the MIMIC intervention pilot trial in a Tanzanian emergency department

Thirty-day outcome	Denominator	Pre-pilot participants (N=275)			Post-pilot participants (N=260)			OR (95 % CI)	*p*
		Total N	Observed n	%	Total N	Observed n	%		
Proportion of participants with AMI alive at 30 days	Participants with confirmed AMI	41	25	61%	29	21	72%	1.67 (0.54-5.46)	0.444
Proportion of participants with AMI taking an antiplatelet at 30 days	Participants with confirmed AMI and 30-day follow-up	41	4	10%	29	15	52%	9.54 (2.49-46.46)	<0.001*
